# Intussusception of the appendix secondary to endometriosis: a case report

**DOI:** 10.1186/s40792-024-02054-x

**Published:** 2024-11-15

**Authors:** Yuta Kawaguchi, Kyoichiro Maekawa, Toshiaki Hashimoto, Mizuki Kitagawa, Shigetoshi Urabe, Emi Yoshimura, Takashi Goto, Mihoko Rikitake, Tohru Iwata

**Affiliations:** 1 Department of Surgery, Japan Organization of Occupational Health and Safety, Nagasaki Rosai Hospital, Nagasaki, Japan; 2 Department of Surgery, Nagasaki University Graduate School of Biomedical Sciences, 1- 7- 1 Sakamoto, Nagasaki, Nagasaki 852-8501 Japan; 3 Department of Gastroenterology, Japan Organization of Occupational Health and Safety, Nagasaki Rosai Hospital, Nagasaki, Japan; 4 Department of Diagnostic Pathology, Japan Organization of Occupational Health and Safety, Nagasaki Rosai Hospital, Nagasaki, Japan

**Keywords:** Intussusception, Appendix, Endometriosis

## Abstract

**Background:**

Intussusception of the appendiceal endometriosis is rare. Although approximately 200 cases of appendiceal intussusception have been reported in the literature, very few have ever been diagnosed preoperatively. Here, we report the case of intussusception of the appendiceal endometriosis with laparoscopic ileocecal resection.

**Case presentation:**

A woman in her 50s presented to the out-patients clinic with epigastric pain lasting for a several month. Contrast-enhanced computed tomography (CT) scanning revealed ileocolic intussusception in which a cecum polypoid mass lesion extended to the hepatic flexure of the ascending colon. A colonoscopy showed a large pedunculated polyp in the cecum. Laparoscopic ileocecal resection was performed. Pathology confirmed an invaginated appendix demonstrating endometriosis implants.

**Conclusions:**

Possible intrinsic causes of intussusception are varied, appendiceal intussusception secondary to endometriosis is extremely rare. Intussusception of the appendix is a rare finding, often mistaken for a polyp. We suggest considering inverted appendix as a differential diagnosis when investigating cecal lesions.

## Introduction

Intussusception is defined as an invagination of one part of the intestine into another section of the bowel [1]. Appendiceal intussusception is an uncommon diagnosis, with an estimated incidence reported by Collins as 0.01% [2]. Approximately 200 cases of appendiceal intussusception have been reported in the literature [3]. In this paper, we present a case of female patient who underwent ileocecal resection presumed diagnosis as cecal tumor, and a histopathological examination of the retrieved specimen revealed appendiceal endometriosis.

## Case report

**Fig. 1:**
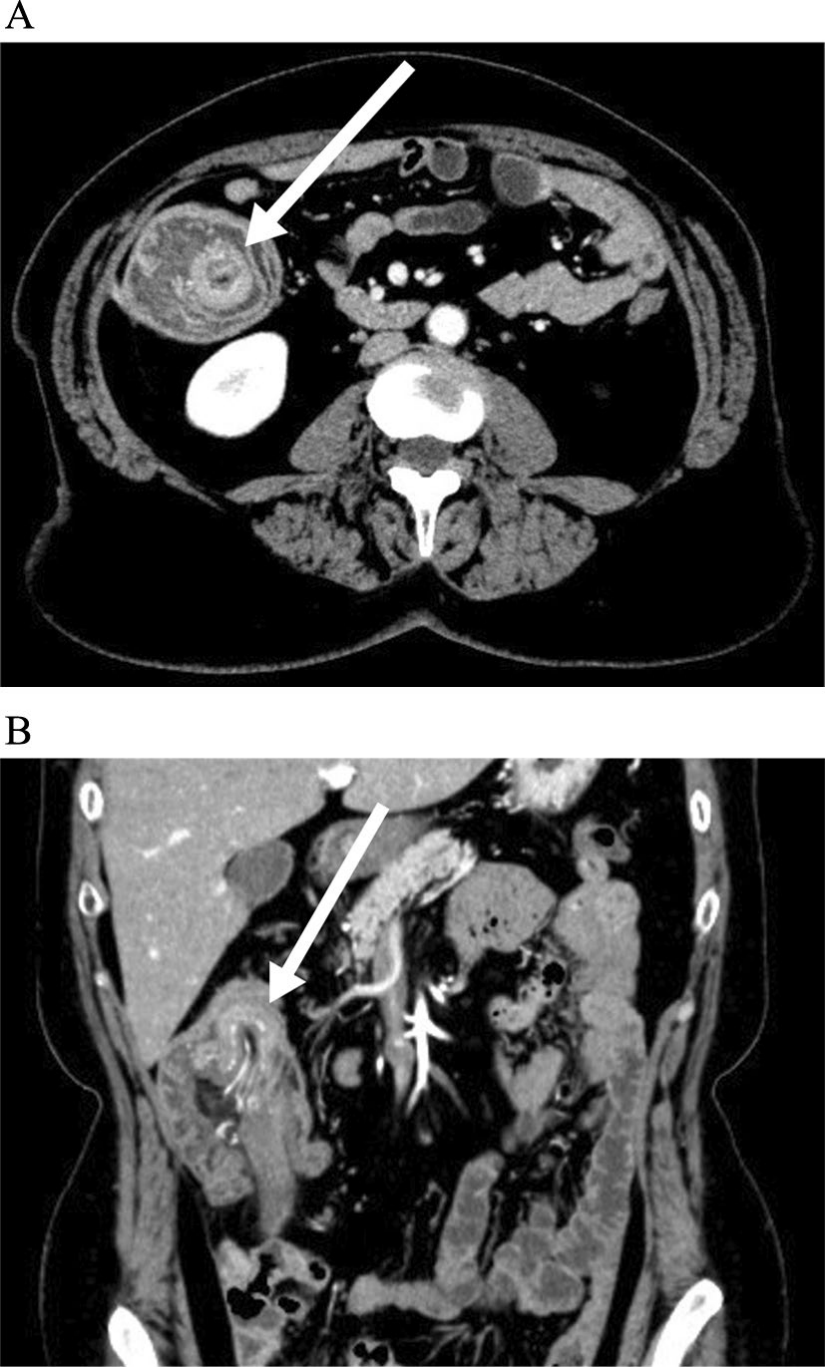
Abdominal computed tomography of abdomen and pelvis with contrast. An ileocolic intussusception is present (arrows) with the cecum polypoid mass lesion telescoping into the ascending colon. **A** Axial view, **B** coronal view

**Fig. 2:**
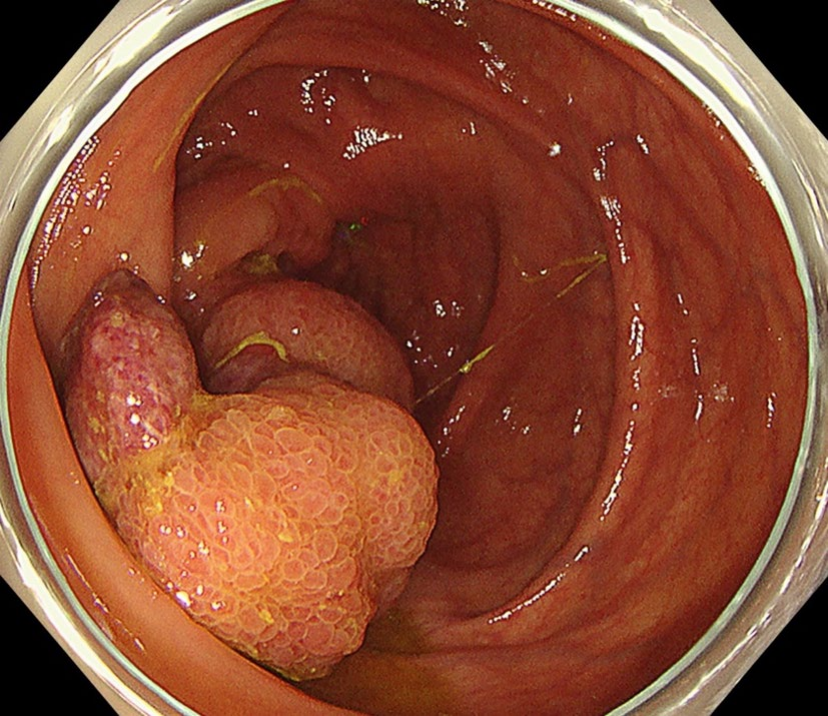
A colonoscopy showed a large pedunculated polyp in the cecum

**Fig. 3:**
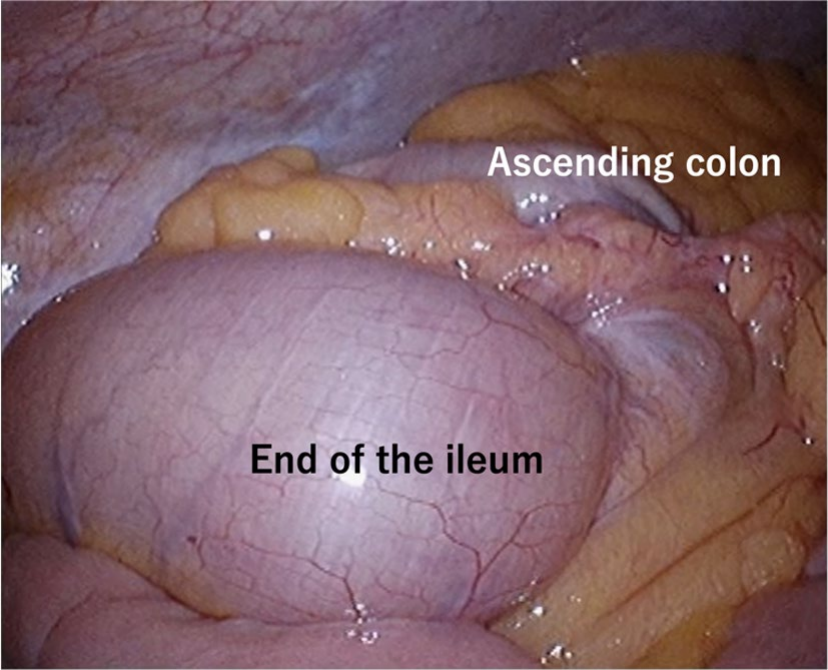
There was no visible appendix in the right iliac fossa

**Fig. 4:**
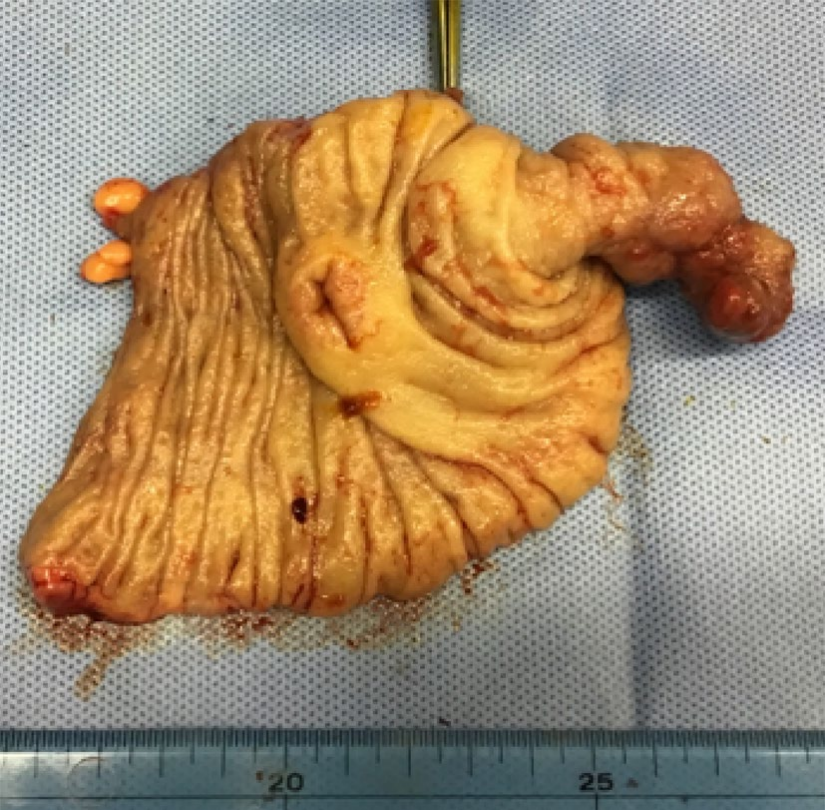
When the specimen was opened, the entire appendix appeared inverted

**Fig. 5:**
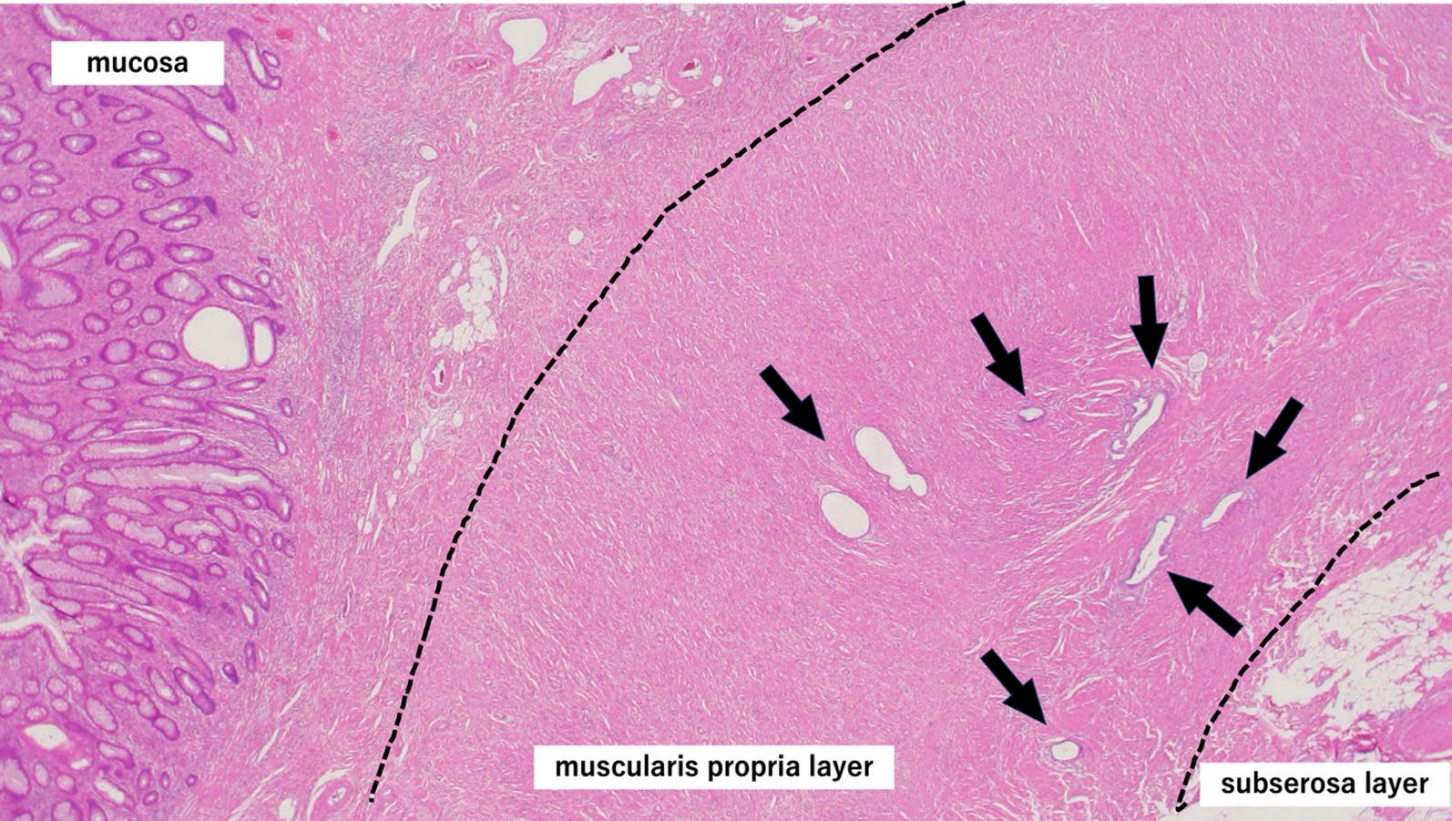
Histopathological exploration showed infiltration of endometrium tissues in the muscularis propria layer of the appendix (arrows, hematoxylin and eosin, 20 ×)

A woman in her 50s presented to gastroenterology out-patients clinic with a several month history of epigastric pain. She had no lack of appetite, fever, nausea or vomiting. The abdominal pain had it blunt character with moderate severity. She had prior history of dysmenorrhea and menorrhagia. Physical examination revealed an abdominal tenderness in the epigastric region. Blood laboratory findings were within normal limits (white blood cells 4.8 × 10^9^/l, red blood cells 4.9 × 10^12^/l, hemoglobin 142 g/l, platelets 300 × 10^9^/l). Upper gastrointestinal endoscopy was performed, but no abnormal findings were found. Contrast-enhanced computed tomography (CT) scanning revealed ileocolic intussusception in which a cecum polypoid mass lesion measuring 4.5 × 2.2 cm extended to the hepatic flexure of the ascending colon (Fig. 1). A colonoscopy was duly organized, we reduced the intussusception by endoscopic manipulation and insufflation. It showed a large pedunculated polyp in the cecum (Fig. 2). On biopsy, this was negative for dysplasia. After discussion with the patient regarding the possibility of cecal cancer, among other diagnoses, she agreed to proceed with a laparoscopic ileocecal resection. During the exploration there was no visible appendix in the right iliac fossa. There were no intraoperative macroscopic findings of endometriosis such as blueberry spots, adhesions or ovary lesions in the abdomen (Fig. 3). When the specimen was opened, the entire appendix appeared inverted (Fig. 4). Histopathological exploration showed infiltration of endometrium tissues in the muscularis propria layer of the appendix. There were no abnormal findings in the mucosa (Fig. 5). Multiple reactive lymph nodes were seen at the attached serosal tissue around the appendix, without any pathological significance. The patient recovered uneventfully and was discharged home. She has since been seen in our clinic and is free of symptoms. She was referred to gynecology colleagues for management of her endometriosis.

## Discussion

Intussusception of the appendix is rare, affecting an estimated 0.01% of the population [4]. Intussusception of the appendix secondary to endometriosis is also extremely rare, with less than 30 cases reported [5]. Additionally, not all cases of appendix intussusception are symptomatic. When symptomatic, the presentation is most frequently chronic [2]. The pathology affects predominantly adults, in particular women [6]. Although the cause of intussusception is unknown, the postulated mechanism for its occurrence is abnormal peristalsis provoked by local irritation [7]. Possible intrinsic causes of intussusception are varied and include foreign body, fecalith, polyp, carcinoid or other neoplasm, mucocele, Crohn's disease, parasites, lymphoid hyperplasia, or endometriosis. The appendix may be completely normal or may harbor a malignancy, endometriosis, or any of the other conditions listed [3].

Intussusception of the appendix is classified into five anatomic types: type I—invagination of the appendiceal tip; type II—the appendiceal tip is more invaginated to the proximal part of the appendix; type III—intussusception begins at the appendiceal base; type IV—retrograde intussusception; type V—complete appendiceal invagination into the cecum [1]. In our case it was the type last mentioned, i.e., complete appendiceal invagination into the cecum.

Although approximately 200 cases of appendiceal intussusception have been reported in the literature, very few have ever been diagnosed preoperatively [8]. In fact, less than 10 cases have been reported in which a preoperative diagnosis had been made; ultra-sound and barium enema were useful in diagnosing many of these [9–13], whereas diagnosis by colonoscopy has been noted in only a select few [8, 14, 15]. An appendiceal intussusception is a rare finding, often mistaken for a polyp. In our patient, the appendiceal intussusception found on the CT scan appeared as a cecal mass. We suggest considering inverted appendix as a differential diagnosis when investigating cecal lesions.

Endometriosis is a condition characterized by the growth of the endometrial tissue outside the uterine cavity. It was initially described by von Rokitansky in 1860 [16]. The reported incidence in pre-menopausal women is in the order of 8–15%. Although the disease classically involves the pelvic organs and pelvic peritoneum, seeding has been observed in surgical scars, around the umbilicus, in the inguinal canal, intestines, bladder, heart and lungs [17]. The exact etiology of endometriosis is unknown, but there are two main theories on its pathogenesis. The transportation theory presumes that endometrial cells are transported to distant sites through surgical manipulation, menstrual shedding via the fallopian tubes or through lymphatic or vascular spread. Alternatively, the metaplastic theory suggests that embryonic coelomic mesothelium dedifferentiates into endometrial tissue in response to inflammation or trauma [18, 19]. The most common symptoms of endometriosis are dysmenorrhea, pelvic pain and infertility but patients can also be asymptomatic [17].

Involvement of the gastrointestinal tract is reported to affect between 3 and 37% of patients with pelvic endometriosis [20, 21]. When endometriosis does involve the gastrointestinal tract it commonly involves the recto-sigmoid (72%), the recto-vaginal septum (13%), small intestine (7%), cecum (3.6%) and the appendix (3%) [22]. Endometriosis of the appendix constitutes a small percentage of all cases of gastrointestinal endometriosis. Collins reported that the rate of appendiceal endometriosis was 0.05% in 71 000 cases of appendectomy [4].

Appendiceal endometriosis is usually asymptomatic [23], but it can mimic appendicitis, perforation, intussusception, or lower gastrointestinal bleeding [24]. Frequently, such symptoms occur at the time of menses [25]. But our patient had intermittent chronic abdominal pain that did not coincide with her menses. While appendiceal endometriosis may have various clinical presentations, without any specific symptoms. It is difficult to make an accurate preoperative diagnosis [26].

In the intestine, endometriosis usually involves the serosa and the subserosa. Sometimes the muscularis propria, the submucosa and the mucosa may be involved especially in symptomatic patients [27]. Appropriate treatment includes resection—both to establish a definitive diagnosis and to alleviate symptoms [28]. Given the nature of endometriosis, patients like ours should be encouraged to follow up with a gynecologist.

## Data Availability

Not applicable.
